# Comparing clinical assessments of pneumonia to bronchoalveolar lavage results in critically ill patients with suspected pneumonia

**DOI:** 10.1093/annalsats/aaoag050

**Published:** 2026-03-16

**Authors:** Caroline F Zhao, Catherine A Gao, Helen K Donnelly, Erin A Korth, Francisco J Martinez, Bridget Giblin, Lesley Pinzon, Rebecca K Clepp, Wan-Ting Liao, Nandita R Nadig, Benjamin D Singer, Richard G Wunderink, Chiagozie I Pickens

**Affiliations:** Department of Internal Medicine, Northwestern University Feinberg School of Medicine, Chicago, IL, United States; Division of Pulmonary and Critical Care Medicine, Northwestern University Feinberg School of Medicine, Chicago, IL, United States; Division of Pulmonary and Critical Care Medicine, Northwestern University Feinberg School of Medicine, Chicago, IL, United States; Division of Pulmonary and Critical Care Medicine, Northwestern University Feinberg School of Medicine, Chicago, IL, United States; Division of Pulmonary and Critical Care Medicine, Northwestern University Feinberg School of Medicine, Chicago, IL, United States; Division of Pulmonary and Critical Care Medicine, Northwestern University Feinberg School of Medicine, Chicago, IL, United States; Division of Pulmonary and Critical Care Medicine, Northwestern University Feinberg School of Medicine, Chicago, IL, United States; Division of Pulmonary and Critical Care Medicine, Northwestern University Feinberg School of Medicine, Chicago, IL, United States; Division of Pulmonary and Critical Care Medicine, Northwestern University Feinberg School of Medicine, Chicago, IL, United States; Division of Pulmonary and Critical Care Medicine, Northwestern University Feinberg School of Medicine, Chicago, IL, United States; Division of Pulmonary and Critical Care Medicine, Northwestern University Feinberg School of Medicine, Chicago, IL, United States; Division of Pulmonary and Critical Care Medicine, Northwestern University Feinberg School of Medicine, Chicago, IL, United States; Division of Pulmonary and Critical Care Medicine, Northwestern University Feinberg School of Medicine, Chicago, IL, United States

**Keywords:** respiratory, infection, diagnosis, intubated

## Abstract

**Rationale:**

While clinical criteria are used to diagnose pneumonia in critically ill patients, rates of concordance between a clinician’s suspicion for pneumonia and a diagnosis of lower respiratory tract infection using bronchoalveolar lavage (BAL) results are undefined.

**Objectives:**

To assess rates of concordance between clinically suspected pneumonia and pneumonia diagnosed by BAL and clinical adjudication in mechanically ventilated patients undergoing BAL for suspected pneumonia, and to identify clinical factors and outcomes associated with diagnostic discordance.

**Methods:**

This was a single-center prospective observational study of intubated, mechanically ventilated patients undergoing BAL for suspected pneumonia. From 2018 to 2022, clinicians were asked to quantify their suspicion for pneumonia on the same day they performed a BAL for the patient with one of the following options: <15%, 30%, 50%, 70%, or >85%. Responses were categorized as low (<15% to 30%), intermediate (50%), or high (70% to >85%) suspicion for pneumonia and compared to diagnoses of pneumonia based on independent adjudication of clinical data plus BAL results.

**Results:**

Among 659 patients, 84% (553/659) were adjudicated to have pneumonia based on chart review and BAL results. Clinicians assigned a low suspicion for pneumonia to 20% (109/553) of patients with an adjudicated diagnosis of pneumonia. Clinicians assigned a high suspicion for pneumonia in 28% (30/106) of patients without pneumonia based on adjudication. Among patients with an adjudicated diagnosis of pneumonia, there were no significant differences in vital signs or laboratory values between those assigned a low suspicion for pneumonia and those assigned a high suspicion for pneumonia. In patients adjudicated to have culture-negative pneumonia (*n* = 117), those assigned a low suspicion for pneumonia, compared to those assigned a high suspicion for pneumonia, had a longer length of stay in the hospital (36 days vs 18 days, *P* = .02) and intensive care unit (ICU) (21 days vs 9 days, *P* = .01).

**Conclusions:**

Overdiagnosis, rather than a missed diagnosis, is the more frequent cause of diagnostic discordance. A low suspicion for pneumonia in patients with an adjudicated diagnosis of culture-negative pneumonia is associated with longer ICU and hospital lengths of stay. There is a need to improve diagnostic accuracy in critically ill patients with suspected pneumonia.

## Introduction

Pneumonia is diagnosed by clinical signs and symptoms of respiratory infection with a radiographic correlate.^1^ However, these criteria can have a specificity as low as 55%, leading to high rates of discordance between the initial and final diagnoses in hospitalized patients with suspected pneumonia.^2-4^ Furthermore, compared to patients who have a correct initial diagnosis, patients with an incorrect initial diagnosis of pneumonia have a higher 30-day mortality.^5^ It is therefore important to identify factors contributing to diagnostic discordance. In critically ill patients who are intubated, diagnostic uncertainty in pneumonia may be heightened due to the difficulty in chest radiograph interpretation of intubated patients, variation in clinical presentation, and occurrence of other syndromes that have overlapping features.^6^ Differences between a clinician’s initial and final diagnosis may impact treatment, hospital length of stay, and decisions to order additional tests. Therefore, there is a need to identify critically ill patients at risk of diagnostic discordance and to understand factors that may influence diagnostic accuracy related to pneumonia in the intensive care unit (ICU).

Importantly, many studies examining diagnostic uncertainty in pneumonia ascertain the diagnosis from *International Classification of Diseases, Tenth Revision* codes and/or clinical notes, which may not capture severe pneumonia or the etiology of pneumonia.^7^ Few studies of clinician diagnostic accuracy have leveraged lower respiratory tract sampling to confirm or exclude pneumonia. We therefore conducted a prospective, observational study in critically ill, mechanically ventilated patients who had a bronchoalveolar lavage (BAL) obtained by their clinical team to further evaluate suspected pneumonia. We chose this study population as these are the patients most likely to be empirically treated with antibiotics for pneumonia. We specifically sought to define the relationship between clinically suspected pneumonia and the presence of lower respiratory tract infection assessed by BAL and physician adjudication.

## Methods

### Study cohort

Patients in this study were enrolled from the Successful Clinical Response to Pneumonia Therapy (SCRIPT) Systems Biology Center. SCRIPT is a National Institutes of Health–funded, single-center, prospective observational study of mechanically ventilated patients with suspected pneumonia (institutional review board #STU00204868).^8-10^ SCRIPT enrolls patients from the Northwestern Memorial Hospital medical ICU (patients with primary cardiac, surgical, or neurological critical illness diagnoses are admitted to other ICUs). Recruitment for SCRIPT began in July 2018, remained active during the severe acute respiratory syndrome coronavirus 2 (SARS-CoV-2) pandemic, and is ongoing.^11^ From 2018 to 2022, 12 779 patients were admitted to the medical ICU and 25% (3226/12 779) were intubated. Of the intubated patients, 57.8% (1865/3226) underwent at least one BAL or non-bronchoscopic BAL (NBBAL). Patients in the medical ICU are screened and approached for enrollment in SCRIPT if the clinical team has ordered a BAL or NBBAL to evaluate suspected pneumonia. In this manuscript, the term “BAL” will also include samples obtained using NBBAL. Clinical testing of all BAL samples includes semi-quantitative culture, multiplex polymerase chain reaction (PCR) testing for common pneumonia pathogens, cell count, and differential.

### Clinician assessment of pneumonia

From July 2018 to November 2022, attending physicians or ICU fellows were approached by a member of the research team and asked to select one of the following options for their clinical suspicion of pneumonia: <15%, 30%, 50%, 70%, or >85%. This question was asked on the day the BAL was performed. Note that this question is not an assessment of pretest probability, as the clinical team already decided to perform a BAL to further evaluate suspected pneumonia. Thus, in all patients there was a non-zero probability of clinically suspected pneumonia. The research team recorded the response of the clinician on a paper form and entered the response into REDCap. Clinicians were not asked to provide rationale or explanation for the pre-BAL probability of pneumonia category they selected.

### Clinical adjudication

The clinician assessment of pneumonia on the day of the BAL was compared to the BAL results paired with adjudication. Note that while pneumonia is a clinical diagnosis, the presence of lower respiratory tract infection is more accurately identified through lower respiratory tract sampling. Therefore our reference standard to diagnose pneumonia was BAL results paired with clinical adjudication. To perform adjudication, 2 physicians independently reviewed the medical record of the enrolled patient to decide if an episode of pneumonia was present.^12^ The physicians specifically reviewed physiological variables, laboratory datapoints, chest radiograph, computed tomography scans, ventilator parameters, clinical documentation, BAL procedure findings, and microbiologic results. Pneumonia was defined as a lower respiratory tract infection diagnosed by positive BAL fluid culture, positive BAL fluid multiplex PCR, or negative BAL fluid culture with a white blood cell differential containing >50% neutrophils and no alternative explanation in a patient with clinical signs and symptoms of pneumonia as determined by the treating physician. If pneumonia was present, the adjudicator classified the pneumonia episode as community-acquired pneumonia (CAP), hospital-acquired pneumonia (HAP), or ventilator-associated pneumonia (VAP) and then selected an etiology of pneumonia. The options for pneumonia etiology were:

1. Viral only: A virus is detected from an upper or lower respiratory tract specimen with evidence of inflammation in BAL fluid.
2. Bacterial–viral coinfection: A virus is detected from an upper or lower respiratory tract specimen and bacteria by positive BAL fluid culture or PCR.
3. Bacterial only: Bacteria are detected from BAL fluid by positive culture or positive PCR with negative viral detections from upper and lower respiratory tract samples or not tested.
4. Culture negative: No viral or bacterial organisms are detected from respiratory specimens via culture or PCR but the BAL fluid white blood cell differential contains >50% neutrophils and no alternative explanation is likely.

If pneumonia was not present, the adjudicator selected “non-pneumonia control” as an option. A non-pneumonia control is a case in which clinical data and respiratory sample results are not consistent with pneumonia (negative BAL culture and BAL fluid differential contains <50% neutrophils). Examples include aspiration pneumonitis, pulmonary hemorrhage, heart failure, or non-pneumonia-induced acute respiratory distress syndrome. For each non-pneumonia control, the adjudicator selected the non-pneumonia cause of respiratory failure. In addition to determining the presence and etiology of pneumonia, the adjudicators also determined the presence of cure versus non-cure at the end of the antibiotic course, and determined an overall clinical outcome at the end of the hospitalization.

Consensus was achieved if the 2 adjudicators selected the same response options for every question in the adjudication worksheet. If at least one discrepancy existed in the answers from the adjudicators, a third adjudicator reviewed the question for which there was discordance (blinded to the answers of the 2 initial adjudicators) and provided a response. If the response was concordant with one of the initial adjudicators, consensus was achieved. If there was a discrepancy in the responses from all 3 reviewers, the question was discussed by the entire adjudication panel.

### Outcomes of interest and statistical analysis

The primary outcome of this study was the percent concordance between the level of suspected pneumonia as assessed by the clinician on the day of the BAL, and the adjudicated diagnosis of pneumonia (based on chart review plus BAL results). We defined diagnostic concordance as the presence of adjudicated pneumonia when clinicians assigned a high suspicion for pneumonia (>50%), or absence of adjudicated pneumonia when clinicians assigned a low (<50%) suspicion for pneumonia. Patients assigned a 50% suspicion for pneumonia were excluded in our analysis of concordance, but included in the other analyses. We analyzed patient-level characteristics and clinical outcomes associated with diagnostic discordance. Analyses were performed using R Statistical Software (version 4.3.1; R Core Team 2023). Continuous variables were reported as medians with first and third quartiles (ie, interquartile range) and compared using Wilcoxon rank-sum test. Categorical variables were compared using Fisher exact test. Missing data were handled via multiple imputations using the *mice* package in RStudio. The code for this project is available at https://github.com/NUSCRIPT/Pneumonia_Probability.

## Results

**Figure 1:**
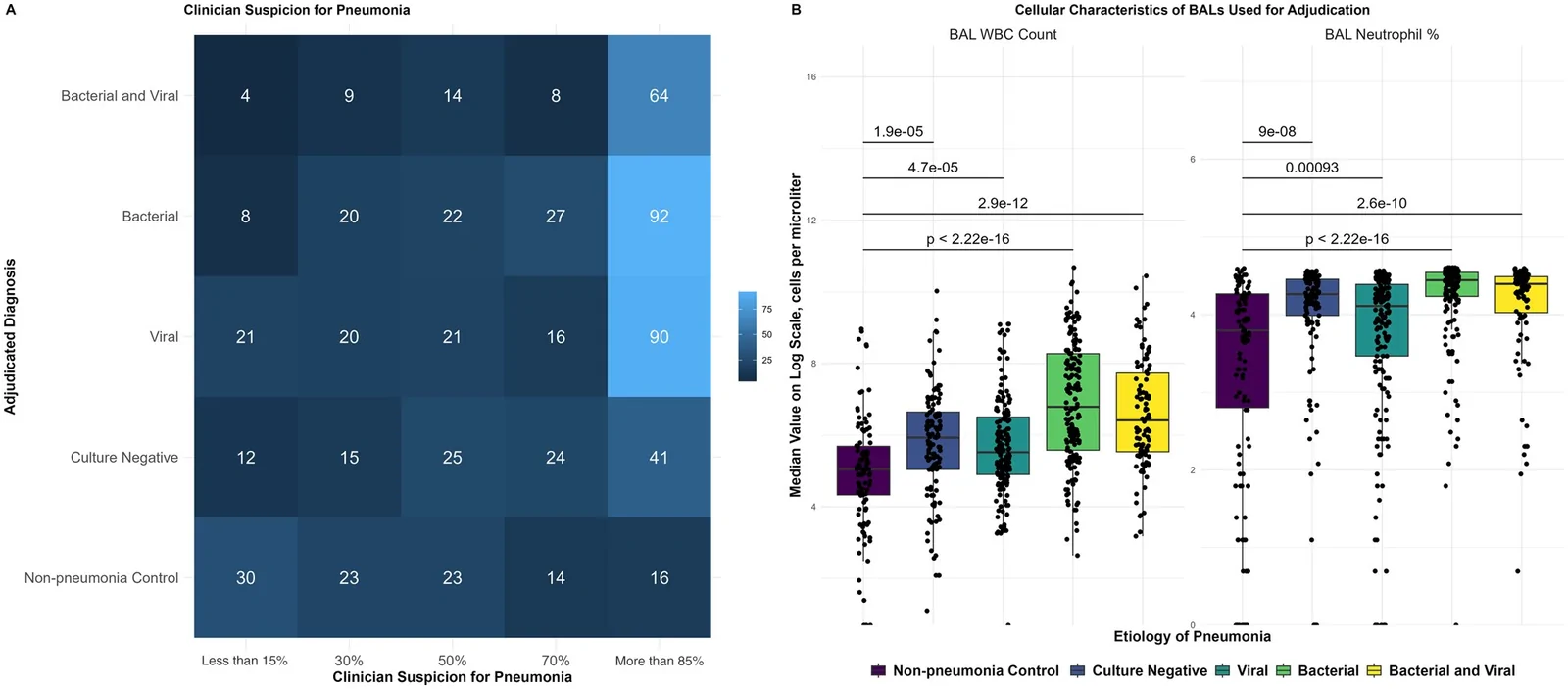
(A) A heatmap of the number of patients in each assigned category for clinician suspicion for pneumonia and the adjudicated diagnosis based on review of clinical data plus BAL results. Each number corresponds to the number of patients. (B) A boxplot of the median BAL leukocyte count and the median percentage of neutrophils for each adjudicated etiology of pneumonia. The ends of the boxplot represent the first and third quartiles (ie, interquartile range) and the horizontal line in the middle of the box represents the median. Abbreviations: BAL, bronchoalveolar lavage; WBC, white blood cell.

**Figure 2:**
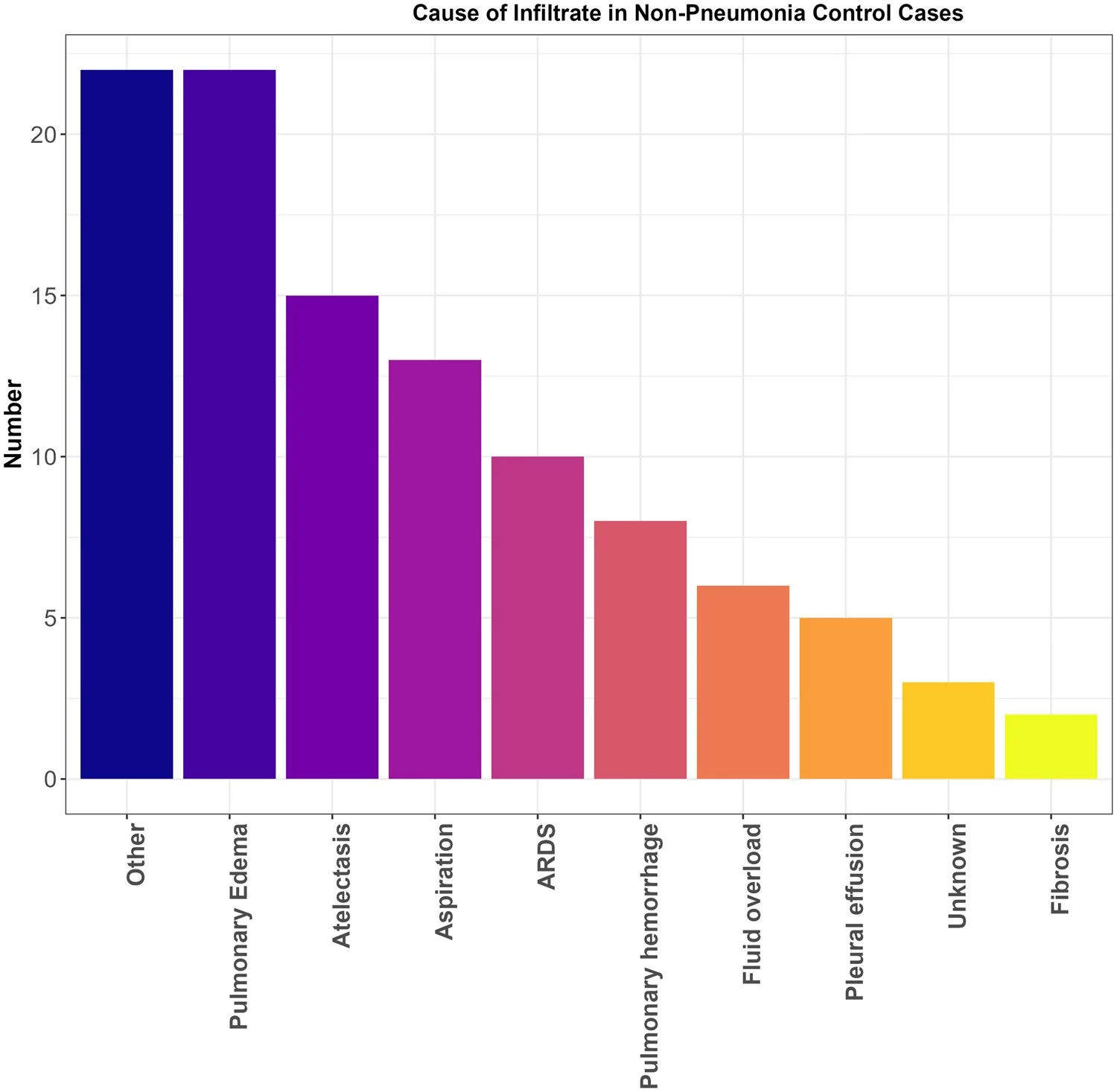
A bar graph depicting the etiologies of respiratory failure for cases that were adjudicated to be non-pneumonia controls. While some patients had multiple causes of respiratory failure, the primary cause was selected by 2 independent adjudicators. The category of “other” represents a variety of causes (eg, bronchiectasis, drug-induced pneumonitis, malignancy). Abbreviation: ARDS, acute respiratory distress syndrome.

**Figure 3:**
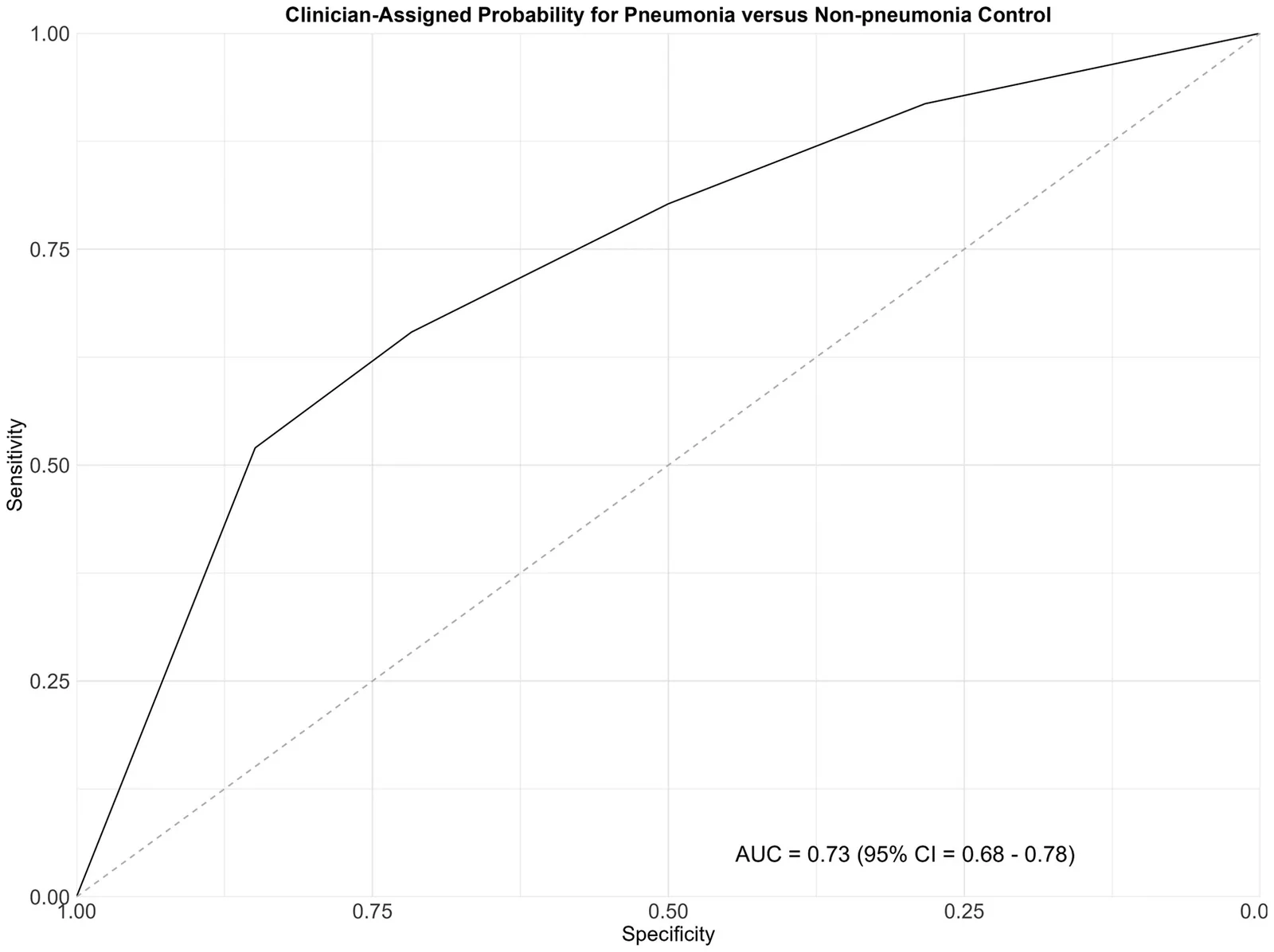
Receiver operating characteristic curve of the diagnostic accuracy of a high clinical suspicion for pneumonia in patients undergoing BAL for suspected pneumonia. Patients with community-acquired pneumonia, hospital-acquired pneumonia, ventilator-associated pneumonia, or culture-negative pneumonia based on clinical adjudication plus BAL results were categorized as “pneumonia.” Patients adjudicated to be non-pneumonia controls were categorized as “no pneumonia.” The predictor variable was a high clinical suspicion for pneumonia (as opposed to a low or intermediate suspicion). Abbreviations: AUC, area under the curve; BAL, bronchoalveolar lavage; CI, confidence interval.

From July 2018 to November 2022, 659 BALs for which a clinician assessment of pneumonia was reported were available for analysis. The clinical characteristics of patients in each level of suspected pneumonia are listed in Table 1. The distribution of suspected pneumonia scores assigned by clinicians on the day of the BAL, compared to the adjudicated diagnosis, is depicted in Figure 1A, and the BAL characteristics for the BALs in each pneumonia category are depicted in Figure 1B. Eighty-four percent (553/659) of the cases were adjudicated as pneumonia. Diagnostic concordance was present in 80% (445/553) of these cases. A low suspicion for pneumonia was assigned to 20% (109/553) of patients later adjudicated to have pneumonia based on BAL and clinical findings. Sixteen percent (106/659) of cases were adjudicated as “non-pneumonia control.” In 50% (53/106) of these cases, a low suspicion for pneumonia was assigned on the day of the BAL. Clinicians assigned a high suspicion for pneumonia in 28% (30/106) of the patients adjudicated to be non-pneumonia controls. For non-pneumonia control cases, the alternative cause of the radiographic infiltrate is listed in Figure 2. Taken together, overdiagnosis in non-pneumonia controls occurred more often than a missed diagnosis in patients adjudicated to have pneumonia based on clinical and BAL results (28% vs 20%, *P* = .05). As culture-negative pneumonia can be a complex diagnosis, we separated these cases out and evaluated the concordance between clinical suspicion for pneumonia in the 66% (436/659) of cases that were adjudicated as viral, bacterial, or viral–bacterial coinfection pneumonia. In 19% (82/436) of these cases, clinicians had a low suspicion for pneumonia, similar to the rate of discordance when culture-negative pneumonia was included. A low suspicion for pneumonia was more frequently assigned to patients with HAP (24.3% [21/146]) compared to patients with CAP (14.4% [54/222]) or VAP (18.4% [34/185]), although this difference did not meet statistical significance (*P* = .06). Overall, using BAL and clinician adjudication as the reference standard, a high clinician suspicion for pneumonia on the day of BAL had a sensitivity of 65%, specificity of 72%, negative predictive value of 28%, and positive predictive value of 92% for differentiating pneumonia from non-pneumonia controls in ICU patients undergoing BAL for suspected pneumonia (Figure 3).

**Table 1:** Characteristics of the study cohort.

Characteristic	Clinician’s suspicion of pneumonia				
	**Less than 15% **(*n* = 75)	**30%** (*n* = 87)	**50%** (*n* = 105)	**70%** (*n* = 89)	**More than 85% **(*n* = 303)
**Clinical suspicion for pneumonia category**	Low		Intermediate	High	
**Pneumonia category, No. (%)**					
** Non-pneumonia control**	30 (40%)	23 (26%)	23 (22%)	14 (16%)	16 (5.3%)
** Community-acquired pneumonia**	10 (13%)	11 (13%)	18 (17%)	20 (22%)	87 (29%)
** Hospital-acquired pneumonia**	21 (28%)	33 (38%)	36 (34%)	32 (36%)	100 (33%)
** Ventilator-associated pneumonia**	14 (19%)	20 (23%)	28 (27%)	23 (26%)	100 (33%)
**Patient demographics**					
** Age, y**	58 (48, 70)	64 (50, 70)	65 (56, 73)	62 (54, 72)	61 (50, 71)
** Female sex, No. (%)**	30 (40%)	46 (53%)	47 (45%)	37 (42%)	107 (35%)
** Body mass index, kg/m^2^**	28 (25, 33)	27 (23, 33)	29 (24, 35)	27 (24, 32)	29 (25, 34)
** Charlson Comorbidity Index score**	4.0 (2.0, 7.0)	4.0 (1.0, 7.0)	4.0 (1.0, 7.0)	5.0 (2.0, 8.0)	3.0 (1.0, 6.0)
** Immunocompromise^a^, No. (%)**	19 (25%)	33 (38%)	29 (28%)	31 (35%)	85 (28%)
**Vital signs and labs, day of BAL**					
** Maximum temperature, °C**	37.5 (37.1, 38.5)	37.4 (37.1, 38.2)	37.8 (37.1, 38.5)	37.7 (37.2, 38.4)	37.9 (37.3, 38.7)
** Maximum heart rate, bpm**	109 (91, 127)	108 (90, 125)	113 (92, 128)	104 (91, 122)	109 (95, 128)
** Average mean arterial pressure, mm Hg**	77 (73, 81)	76 (72, 82)	76 (72, 84)	74 (71, 81)	77 (73, 84)
** Average PaO_2_ to FiO_2_ ratio**	279 (173, 353)	209 (165, 280)	222 (166, 316)	213 (168, 288)	190 (148, 265)
** Average plateau pressure, cm H_2_O**	21.0 (17.5, 24.7)	23.0 (17.5, 28.3)	21.2 (18.0, 26.8)	21.7 (17.5, 27.5)	24.0 (19.0, 28.7)
** Maximum peripheral leukocyte count, cells/μL**	14 (9, 21)	13 (8, 18)	12 (8, 17)	13 (9, 19)	12 (8, 19)
** Average hemoglobin, g/dL**	9.40 (8.05, 11.10)	9.20 (7.90, 10.90)	8.70 (7.53, 10.85)	8.90 (8.00, 10.70)	9.70 (8.20, 12.10)
** Average albumin, g/dL**	2.75 (2.40, 3.30)	2.90 (2.50, 3.10)	2.80 (2.40, 3.20)	2.90 (2.60, 3.20)	2.90 (2.40, 3.30)
**Clinical outcomes**					
** Hospital length of stay, d**	22 (9, 37)	26 (14, 46)	26 (19, 37)	22 (13, 40)	24 (16, 39)
** Total ICU length of stay, d**	12 (5, 27)	19 (7, 34)	18 (10, 29)	13 (8, 23)	16 (8, 30)
** In-hospital mortality, No. (%)**	33 (44%)	47 (54%)	45 (43%)	33 (37%)	119 (39%)

All values are from the day of the BAL procedures. Data represent median (Q1, Q3) unless otherwise indicated.

Abbreviations: BAL, bronchoalveolar lavage; bpm, beats per minute; FiO_2_, fractional inspired oxygen; ICU, intensive care unit; PaO_2_, partial pressure of arterial oxygen.

a Immunocompromise was defined as any condition or medication causing a patient to have a weakened immune system, including organ transplant on immunosuppressive medications, autoimmune disease, prolonged steroid usage, and malignancy.

**Figure 4:**
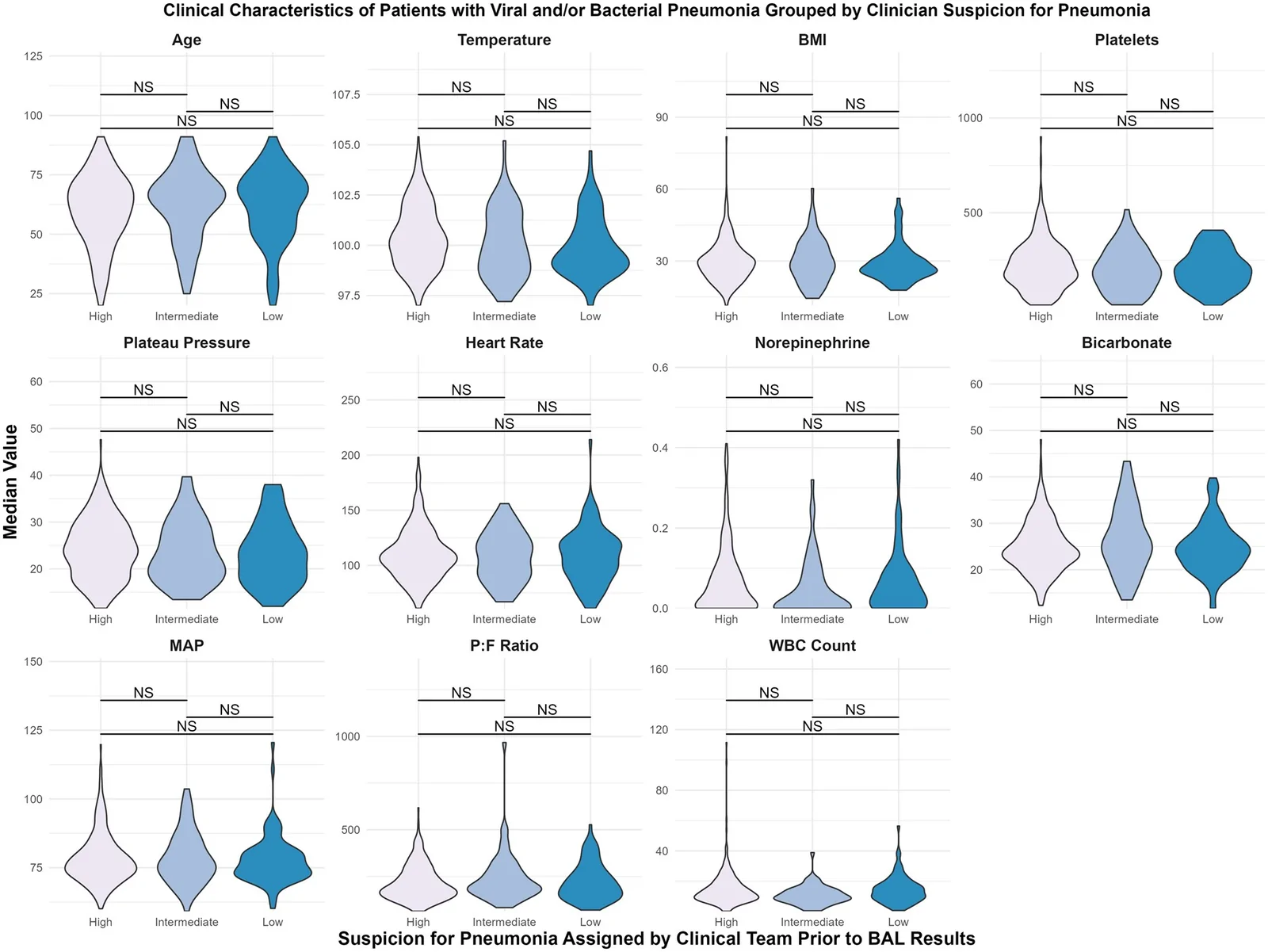
Violin plot for the median values of clinical characteristics for patients adjudicated to have viral and/or bacterial pneumonia are grouped by high, intermediate, and low levels of clinical suspicion for pneumonia on the day of the BAL. NS denotes a *P* value >.05. Abbreviations: BAL, bronchoalveolar lavage; BMI, body mass index; MAP, mean arterial pressure; P:F, ratio of arterial oxygen partial pressure to fractional inspired oxygen; Plat., plateau; WBC, white blood cell.

**Figure 5:**
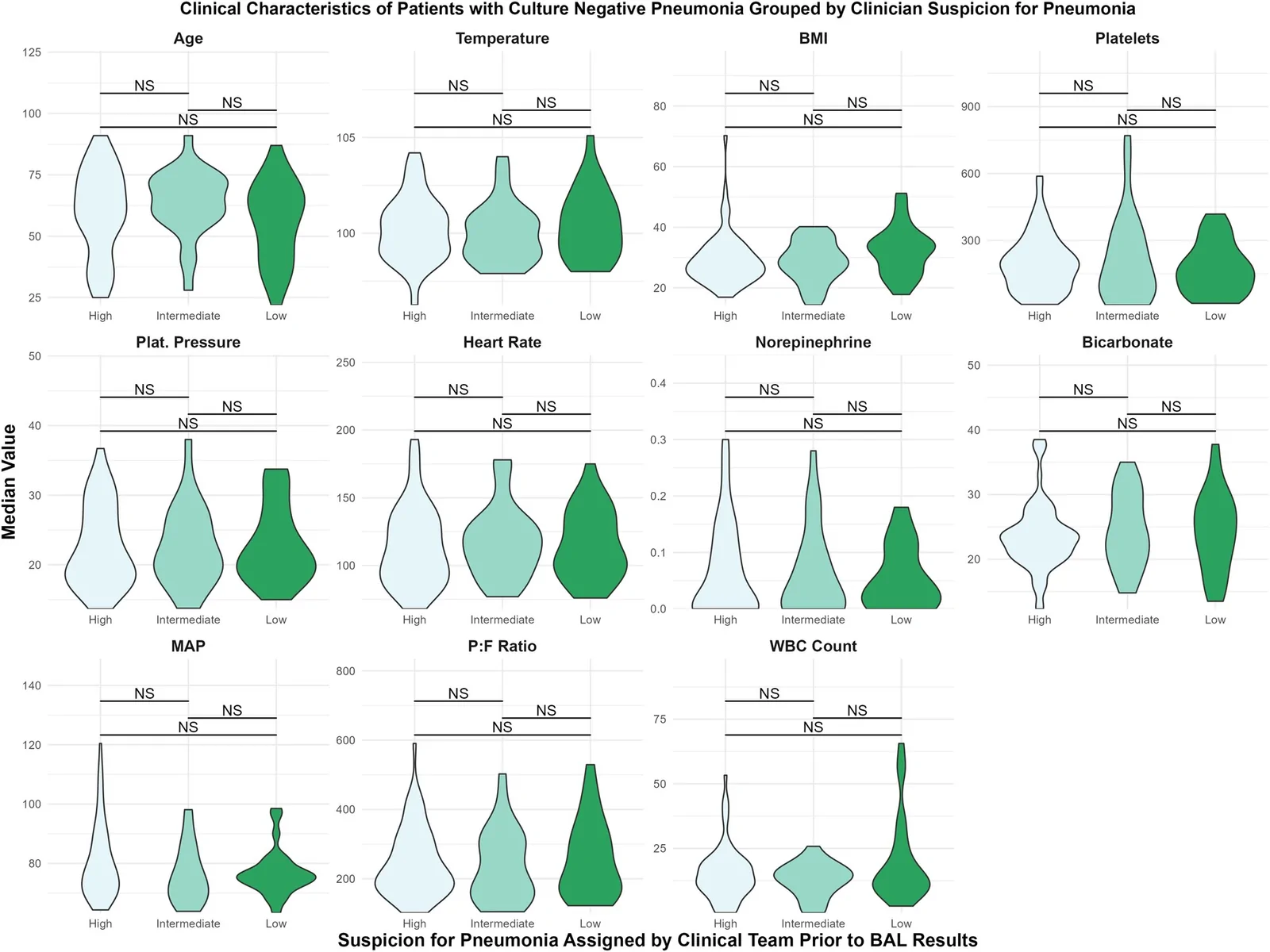
The median values of clinical characteristics for patients adjudicated to have culture-negative pneumonia are grouped by high, intermediate, and low levels of clinical suspicion for pneumonia on the day of the BAL. NS denotes a *P* value >.05. Abbreviations: BAL, bronchoalveolar lavage; BMI, body mass index; MAP, mean arterial pressure; P:F, ratio of arterial oxygen partial pressure (PaO_2_ in mmHg) to fractional inspired oxygen; Plat., plateau; WBC, white blood cell.

**Figure 6:**
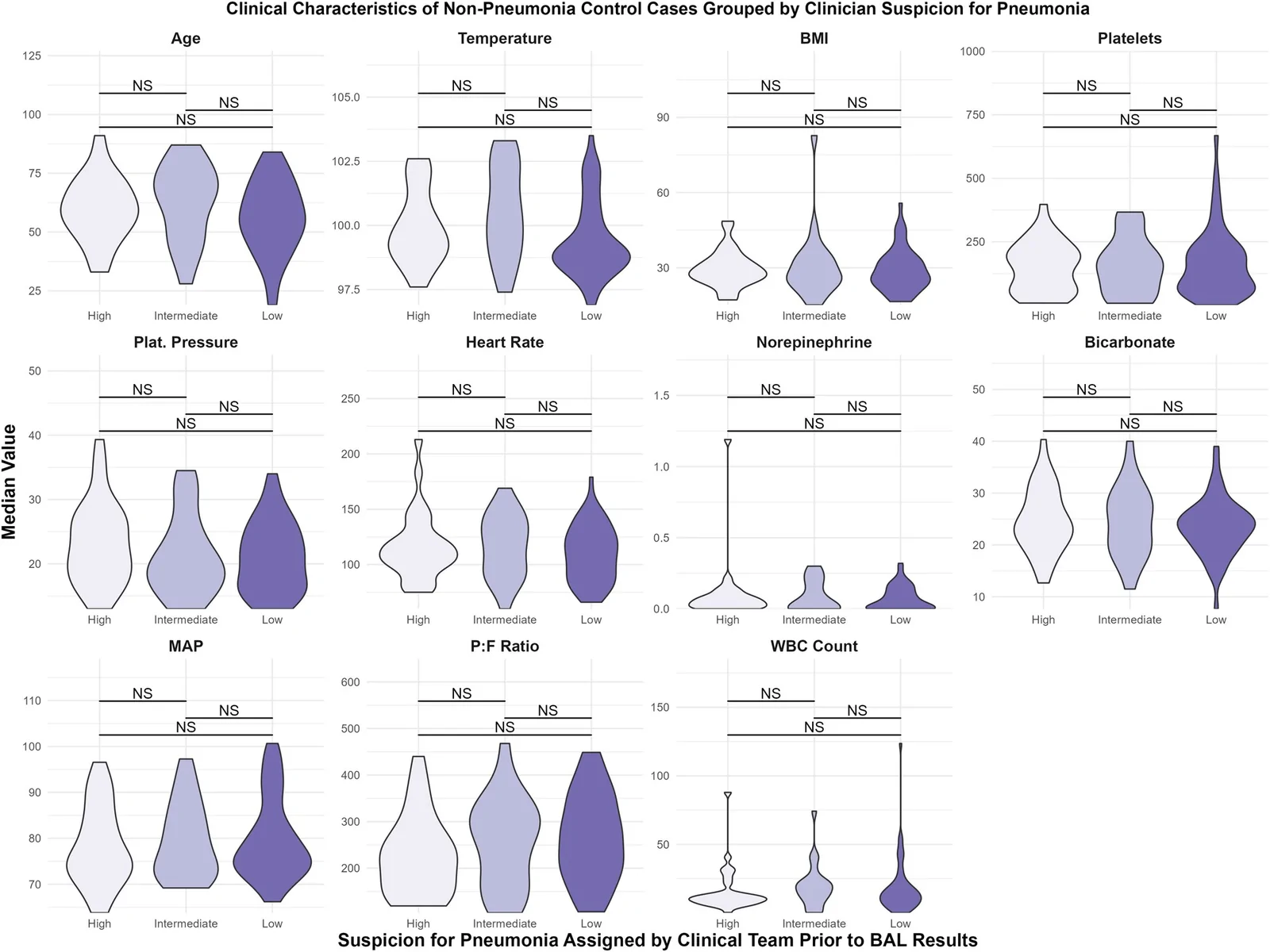
The median values of clinical characteristics for patients adjudicated to be non-pneumonia controls are grouped by high, intermediate, and low levels of clinical suspicion for pneumonia on the day of the BAL. NS denotes a *P* value >.05. Abbreviations: BAL, bronchoalveolar lavage; BMI, body mass index; MAP, mean arterial pressure; P:F, ratio of arterial oxygen partial pressure (PaO_2_ in mmHg) to fractional inspired oxygen; Plat., plateau; WBC, white blood cell.

We then examined the relationship between clinical characteristics and diagnostic discordance. In patients with an adjudicated diagnosis of viral and/or bacterial pneumonia, there were no differences in demographics, vital signs, or laboratory values between those assigned a low, intermediate, or high suspicion for pneumonia on the day of the BAL (Figure 4). Similarly, for patients with an adjudicated diagnosis of culture-negative pneumonia and for non-pneumonia control cases, there were no differences in clinical parameters between those assigned a low, intermediate, or high suspicion for pneumonia prior to the BAL (Figures 5 and 6). We also evaluated differences in clinical outcomes based on the suspicion for pneumonia assigned by the clinician team on the day of the BAL. In patients adjudicated to have culture-negative pneumonia, those who were assigned a low suspicion for pneumonia had a longer ICU and hospital length of stay compared to those assigned a high suspicion for pneumonia. Similarly, cases adjudicated as non-pneumonia controls who were assigned a high suspicion for pneumonia had a longer length of hospital stay compared to those assigned a low suspicion for pneumonia (Table 2).

**Table 2:** Clinical outcomes of patients grouped by the assigned suspicion of pneumonia and the adjudicated, BAL-confirmed diagnosis of pneumonia.

Outcome	Clinician suspicion for pneumonia on day of BAL			*P* value^a^
	High (*n* = 30)	Intermediate (*n* = 23)	Low (*n* = 53)	
**Adjudicated diagnosis of pneumonia based on chart review and BAL results**				
**Non-pneumonia controls**				
** Hospital length of stay, d**	23 (14, 40)	24 (14, 32)	19 (8, 33)	.2
** Total ICU length of stay, d**	14 (8, 22)	14 (7, 25)	7 (5, 18)	.0026
** Hospital discharge status, No. (%)**				.2
** Alive**	19 (63%)	9 (39%)	32 (60%)	
** Died**	11 (37%)	14 (61%)	21 (40%)	
**Bacterial pneumonia**				
** Total hospital length of stay, d**	24 (15, 42)	29 (20, 38)	22 (12, 44)	.5
** Total ICU length of stay, d**	15 (8, 32)	18 (10, 33)	12 (7, 28)	.3
** Hospital discharge status, No. (%)**				.3
** Alive**	117 (61%)	25 (69%)	21 (51%)	
** Died**	74 (39%)	11 (31%)	20 (49%)	
**Viral pneumonia**				
** Hospital length of stay, d**	27 (18, 38)	31 (21, 52)	27 (12, 40)	.3
** Total ICU length of stay, d**	19 (12, 31)	21 (16, 45)	24 (12, 40)	.14
** Hospital discharge status, No. (%)**				.12
** Alive**	63 (59%)	10 (48%)	17 (41%)	
** Died**	43 (41%)	11 (52%)	24 (59%)	
**Microbiology-negative pneumonia**				
** Hospital length of stay, d**	18 (10, 32)	23 (14, 35)	36 (19, 46)	.018
** Total ICU length of stay, d**	9 (6, 20)	13 (8, 20)	21 (10, 28)	.010
** Hospital discharge status, No. (%)**				.2
** Alive**	41 (63%)	16 (64%)	12 (44%)	
** Died**	24 (37%)	9 (36%)	15 (56%)	

Data represent median (Q1, Q3) unless otherwise indicated.

Abbreviations: BAL, bronchoalveolar lavage; ICU, intensive care unit.

a Kruskal–Wallis rank-sum test; Pearson χ^2^ test.

## Discussion

Our results demonstrate that in critically ill, mechanically ventilated patients undergoing BAL for suspected pneumonia, substantial discordance between the clinical suspicion for pneumonia and BAL-based, adjudicated diagnosis of pneumonia exists. For the entire population, overdiagnosis of pneumonia in patients adjudicated to be non-pneumonia controls, compared to a missed diagnosis in patients adjudicated to have pneumonia, is the more common cause of discordance. This may be due to the non-specificity of clinical signs and symptoms in pneumonia in critically ill patients. Given this finding, and the associated implications for therapy (potential antibiotic overuse), additional testing with a diagnostic tool like BAL should be considered in ICU patients with suspected pneumonia.

In this study, the BAL findings were incorporated into an adjudication protocol to determine the presence of pneumonia. A recent study by Soper  et al. also used a combination of clinical signs and BAL findings to improve diagnostic accuracy in patients with pneumonia, focusing specifically on VAP.^13^ The authors used evidence-based likelihood ratios for individual clinical parameters and respiratory culture results to calculate a Bayesian probability of VAP. Importantly, the authors found high rates of clinician overestimation of VAP. As we did not systematically collect all the clinical variables (presence of tracheal secretions, specific type of infiltrate on chest radiograph) used by Soper  et al., we were unable to apply their published framework to our cohort. However, future studies should compare the diagnostic accuracy of this innovative Bayesian model to BAL-based diagnoses of pneumonia.

While overdiagnosis of pneumonia was common in our cohort, it was surprising that 60% of patients who were assigned a less than 15% suspicion for pneumonia were adjudicated to have pneumonia. This highlights the difficulty of using clinical parameters alone to diagnose pneumonia, and also suggests that suspicion for pneumonia may be influenced by variables other than routine clinical parameters. Our study draws attention to the lack of objective diagnostic tools for pneumonia. Even a single clinical sign or an abnormal radiograph has a low, but finite, association with VAP.^14^ Clinicians may overlook a diagnosis of pneumonia in patients with ventilated HAP or VAP, or those transferred to the ICU with sepsis, due to the presence of alternative sources of infection in hospitalized patients.^15^

Note that our cohort was comprised of patients undergoing BAL to evaluate some degree of clinically suspected pneumonia. Indeed, 84% of patients undergoing a clinically indicated BAL in this study were adjudicated to have pneumonia. Of note, our adjudication did not account for fungal pneumonia, which may have represented a minority of cases. This high prevalence of pneumonia can be attributed to our enrollment criteria and the fact that our study spanned the SARS-CoV-2 pandemic. The prevalence of pneumonia would likely be lower in a general cohort of ventilated patients in the ICU, which may have increased the rates of diagnostic concordance (ie, if there are no clinical signs or symptoms of pneumonia and the clinician has no suspicion for pneumonia, the adjudicated diagnosis might often be “no pneumonia”). We chose to focus on patients in whom there is some clinical suspicion for pneumonia, because these patients are almost always treated with empirical antibiotics until additional testing sufficiently rules out pneumonia. If microbiologic testing is not pursued, patients may be treated for clinically diagnosed pneumonia with a full course of antibiotics.

We add to the existing literature by demonstrating that a low suspicion for pneumonia in patients adjudicated to have culture negative pneumonia is associated with longer lengths of hospital and ICU stays. Culture-negative pneumonia is a complex diagnosis, and the optimal treatment is unknown. It is possible that despite our adjudicated diagnosis of culture-negative pneumonia, some of these patients had non-infectious etiologies of respiratory failure that were not responsive to antibiotics nor easily reversible, leading to a longer ICU and hospital stay. In contrast, in patients adjudicated to be non-pneumonia control cases, a low suspicion for pneumonia, compared to patients assigned an intermediate or high suspicion for pneumonia, was associated with a shorter ICU length of stay. This patient group may represent those with a clear, alternative diagnosis, which could be associated with earlier initiation of appropriate management.

This study has limitations. First, the time period of the study included the SARS-CoV-2 pneumonia pandemic, which likely impacted clinical suspicion of pneumonia. It is possible that without the presence of a pneumonia pandemic, clinical suspicion for pneumonia would be lower in some patients. However, this does not explain the 20% of cases in which clinicians had a low suspicion for pneumonia in patients adjudicated to have pneumonia based on clinical and BAL data. A second limitation is that this was a single-center study, which limits its generalizability to other patients and institutions. In addition, clinical estimates were made at the time of BAL performance, potentially leading to a low estimated probability since a highly diagnostic test was pending and no clinical decision based on that estimate was required.

Despite these limitations, our study highlights the insufficiency of diagnosing pneumonia in critically ill patients with clinical factors alone. As such, if circumstances allow for it, our findings support BAL as a confirmatory test for the diagnosis of pneumonia in critically ill patients, both to avoid a missed diagnosis and to ensure appropriate treatment. Future studies should evaluate adjunctive tests, biomarkers, and assessments that can add accuracy to the clinical diagnosis of pneumonia in ICU patients.

## Supplementary Material

aaoag050_Supplementary_Data

## Data Availability

The code for this project is available at https://github.com/NUSCRIPT/Pneumonia_probability.git, and a significant portion of the dataset is available on PhysioNet at https://physionet.org/content/script-carpediem-dataset/1.8.0/.1.
